# Layer-by-layer assembled polymeric thin films as prospective drug delivery carriers: design and applications

**DOI:** 10.1186/s40824-018-0139-5

**Published:** 2018-09-26

**Authors:** Sohyeon Park, Uiyoung Han, Daheui Choi, Jinkee Hong

**Affiliations:** Department of Chemical and Biomolecular Engineering, College of Engineering Yonsei University, 50 Yonsei Ro, Seodaemun Gu, Seoul, 038722 Republic of Korea

**Keywords:** Layer-by-layer assembly, Multilayer thin films, Controlled drug delivery, Surface modification

## Abstract

**Background:**

The main purpose of drug delivery systems is to deliver the drugs at the appropriate concentration to the precise target site. Recently, the application of a thin film in the field of drug delivery has gained increasing interest because of its ability to safely load drugs and to release the drug in a controlled manner, which improves drug efficacy. Drug loading by the thin film can be done in various ways, depending on type of the drug, the area of exposure, and the purpose of drug delivery.

**Main text:**

This review summarizes the various methods used for preparing thin films with drugs via Layer-by-layer (LbL) assembly. Furthermore, additional functionalities of thin films using surface modification in drug delivery are briefly discussed. There are three types of methods for preparing a drug-carrying multilayered film using LbL assembly. First methods include approaches for direct loading of the drug into the pre-fabricated multilayer film. Second methods are preparing thin films using drugs as building blocks. Thirdly, the drugs are incorporated in the cargo so that the cargo itself can be used as the materials of the film.

**Conclusion:**

The appropriate designs of the drug-loaded film were produced in consideration of the release amounts and site of the desired drug. Furthermore, additional surface modification using the LbL technique enabled the preparation of effective drug delivery carriers with improved targeting effect. Therefore, the multilayer thin films fabricated by the LbL technique are a promising candidate for an ideal drug delivery system and the development possibilities of this technology are infinite.

## Background

Recently, as the importance of the life extension and the quality of life is more appreciated, biotechnological advances in the field of biomedical science are being increasingly reported. Particularly, research on drug delivery systems has been recognized as one of the most significant challenges in biomedical science [1]. In general, research studies on drug delivery systems focus on maintaining a minimum concentration of drugs in the blood and minimizing drug toxicity in vivo [1–3]. In view of these efforts, new approaches that allow self-controlled drug release are required. Among the various approaches, the application of thin films to drug delivery systems has emerged due to the variety of materials that can be used for preparing the films [4]. Thin films can be fabricated with a polymer, as well as with various functional nanomaterials or drugs. Therefore, thin films allow effective incorporation and a controlled drug release [5, 6].

As new thin-film manufacturing technologies emerge, surfaces that enable localized and precise controlled release of active therapeutics can be fabricated [7, 8]. Strategies for the fabrication of ultrathin film devices include the Langmuir–Blodgett method [9, 10], self-assembled monolayer techniques [11, 12], and layer-by-layer (LbL) assembly [13–22]. LbL assembly is most suited for the fabrication of films used for drug delivery because it imposes no restrictions on the size or shape of the substrate and does not require high temperature or pressure. In the LbL assembly process, multilayer films are deposited onto the surface of the substrate via alternate adsorption of the interacting materials. A variety of materials, including polyelectrolytes, micelles, graphene oxide (GO), nanoparticles, and proteins can be used as building blocks for LbL-assembled multilayer films [23–26]. The materials interact with each other via driving forces such as electrostatic interactions, hydrogen bonds, covalent bonds, and bio-specific interactions. These properties allow the controlled release of the drug, depending on the materials used in the particular multilayer film, containing the drug. Thus, the LbL technique can be considered as the most appropriate method for preparing nano-multilayer films incorporated with therapeutic molecules [27, 28].

There are several factors that need to be considered for the effective incorporation and drug release by the thin films. First, the potential in vitro and in vivo toxicity of the therapeutic molecules and the film material should be recognized before preparing the films incorporated with the specific drug. This is to ensure that the appropriate material has been selected for film preparation, and also to determine the right concentration of the drug that is to be delivered [29, 30]. The second concern is the stability of the thin film, which is to be incorporated with the drug; thin films carrying drugs need to be both chemically and physically stable [31]. To improve stability, a variety of interactions and crosslinking chemistry are used for loading drugs onto pre-fabricated films. Additionally, drugs can be directly used as building blocks for film preparation. Furthermore, drugs can be incorporated in cargoes such as nanoparticles, and these cargoes can be often used as film materials [32–39]. The third consideration is the precise targeting of drugs to the target sites. Stimuli-responsive polymers relating to environment of the target areas are frequently used as film materials to achieve precise drug targeting [40, 41]. Drug-incorporated nanoparticles, exhibit both precise drug targeting and controlled drug release by surface modifications, achieved by using certain coatings. The fourth consideration is the release period and the rate of drug release [42, 43]. Drug release should occur in the target site without suffering losses during the process of delivery. Additionally, the drug release kinetics should be precisely manipulated to ensure a sustained release instead of a burst release, keeping in mind the dose and the target site of the drug. This control can be achieved by controlling the degradation rate of the film, depending on the drug to be incorporated, the film material and the fabrication method for film preparation [44].

## Preparation of drug-incorporated films

### Loading drugs directly onto pre-fabricated thin films

The simple immobilization of drugs onto the surface of the film is the easiest approach for improving the controlled-release systems in biomedical implants, tissue engineering, and targeted drug delivery systems. There are several strategies for immobilization of drugs onto the film surface. In these strategies, immobilization is reported to occur by simple adsorption or via various driving forces including electrostatic interactions, hydrogen bonds, hydrophobic interactions, and van der Waals interactions [10, 45, 46].

A typical example of use of physical immobilization to load the drug onto certain films without chemical usage was suggested [47]. They fabricated polylysine/hyaluronic acid (PLL/HA) multilayer films via LbL assembly, where PLL and HA were used as the polycation and polyanion, respectively. The PLL/HA multilayer film acted as a reservoir for paclitaxel (Taxol), which was embedded on top of it. The paclitaxel molecules easily diffused across the entire section of the (PLL/HA)60 film, and absorption increased proportionately with the thickness of the PLL/HA multilayer film. Additionally, the concentration of paclitaxel within the film depended on the initial concentrations of paclitaxel in the solution during deposition. The drug content in the PLL/HA films could be finely tuned over a large concentration range [47].

**Fig. 1:**
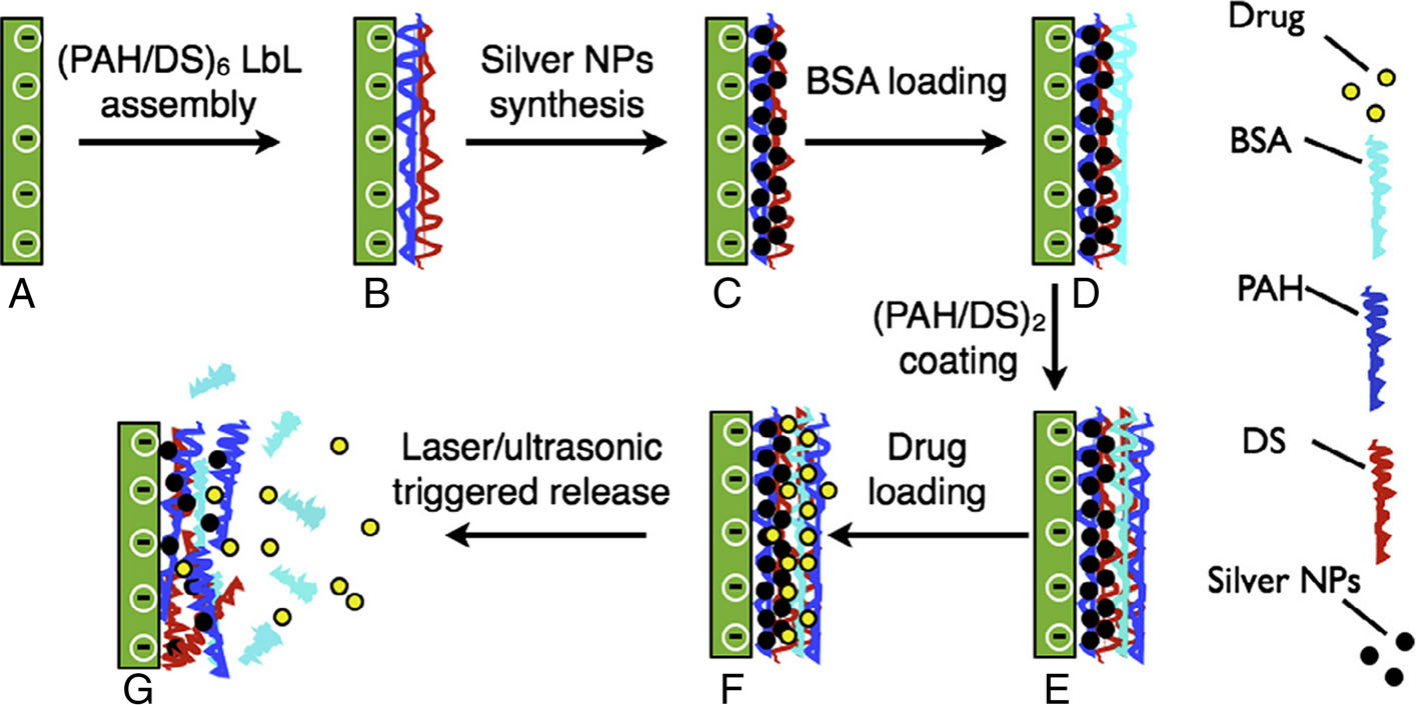
Schematic showing our research methodology for the fabrication of PEM films for remotely activated drug and protein delivery. **a** glass substrate; **a**–**b**, LbL deposition; **b**–**c**, silver NP synthesis; **c**–**d**, BSA loading; **d**–**e**, additional (PAH/DS) layer deposition; **e**–**f**, CH loading; **f**–**g**, remotely activated release. (Reprinted with permission from Ref. [48]. Copyright 2010, Elsevier)

**Scheme 1:**
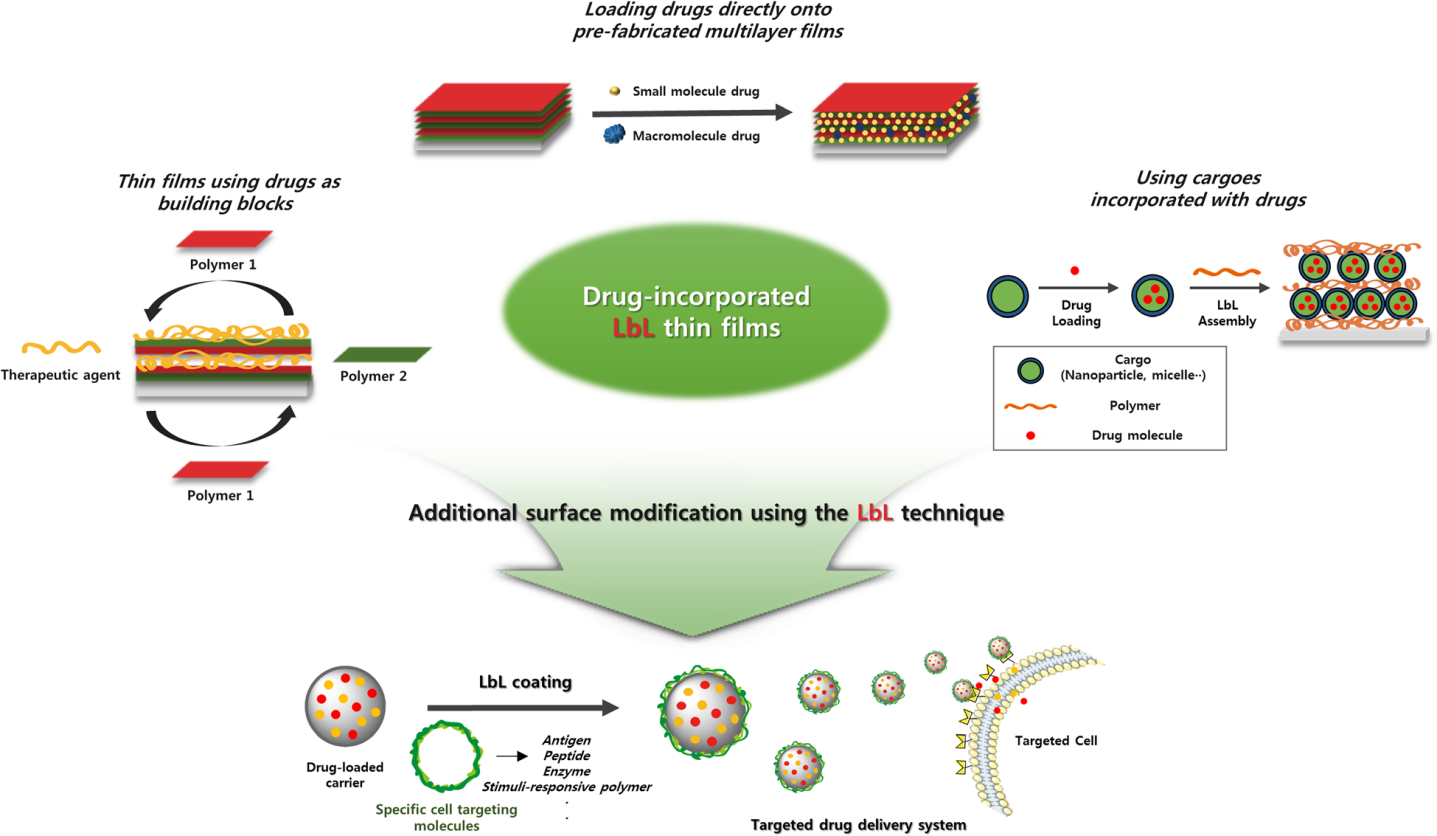
Illustration showing drug delivery system using LbL assembly multilayer film

In addition, LbL assembly using poly(allylamine hydrochloride) (PAH) and dextran sulfate (DS) was designed by alternate adsorptions onto a glass substrate, followed by nanoparticle synthesis via the polyol reduction method described in Fig. 1. Bovine serum albumin (BSA) was used as the model protein, which was immobilized into the polymeric network of the film using electrostatic interactions, hydrogen bonds, and hydrophobic interactions. Following BSA adsorption, two bilayers of PAH/DS were added to the polyelectrolyte multilayers (PEMs) to maintain the negative surface charge of the film. The negative charge would favor the incorporation of the low molecular weight water-soluble drug ciprofloxacin hydrochloride (CH) into the film via electrostatic interactions [48] (Scheme 1).

**Fig. 2:**
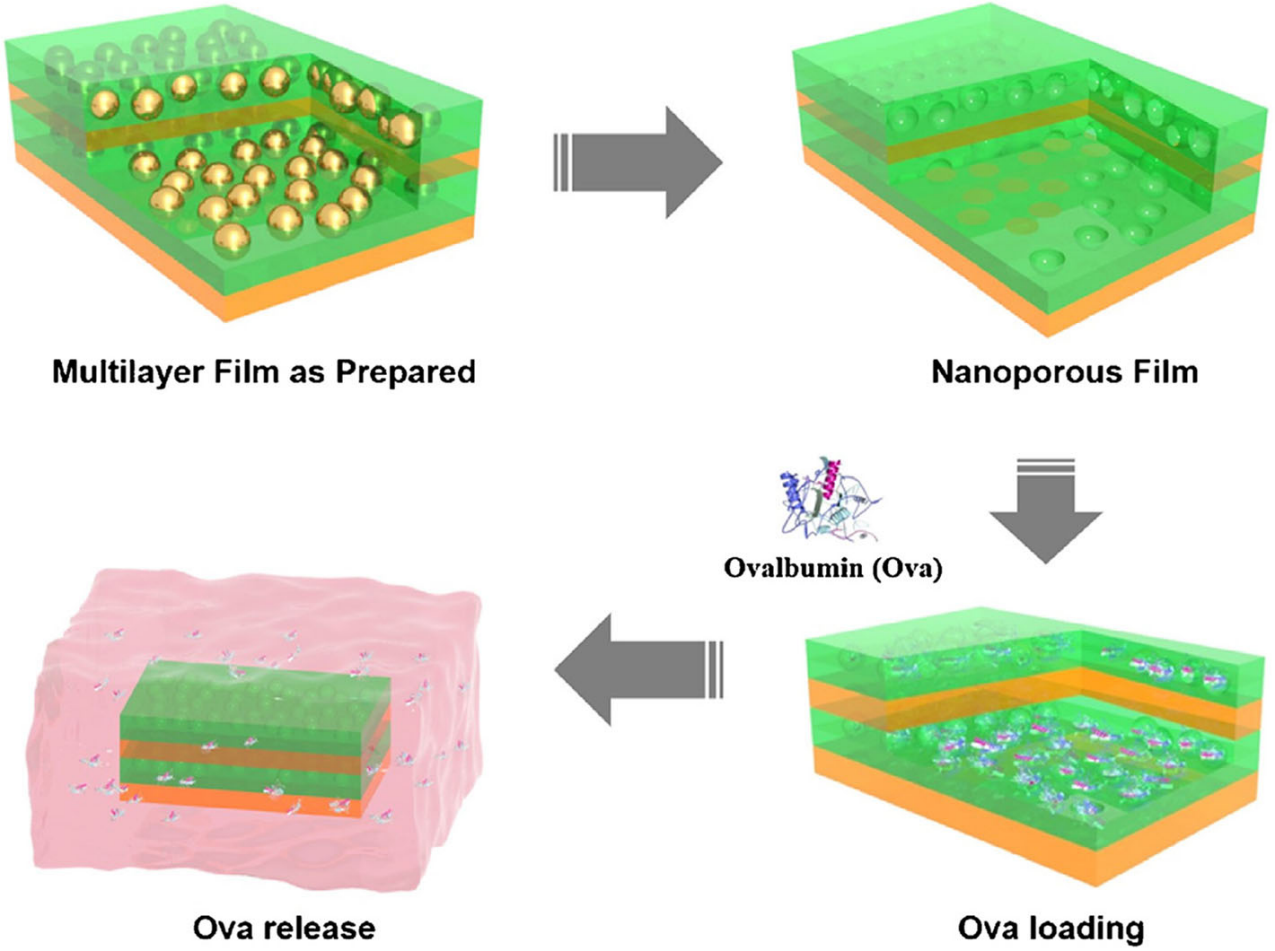
Schematic illustration of the LbL assembly method using BPEI, HA, and gold NPs. General strategy for the preparation of the (BPEI/HA/gold NPs/HA)n (*n* = number of tetralayers) structure-based nanoporous films, and subsequent ova release under model physiological conditions. (Reprinted with permission from Ref. [49]. Copyright 2016, Elsevier)

Moreover, a new route that induces nano-porosity within LbL-assembled multilayer thin films, has been reported for protein delivery. Multilayer thin films were prepared by sequential adsorption of the branched poly (ethylene imine) (BPEI) polycation, polyanionic HA and cationic AuNPs via electrostatic interactions (Fig. 2). AuNPs embedded in the multilayer thin film structure, were easily dissolved using an aqueous cyanide solution to generate the nanoporous film. The incorporation of ovalbumin (ova) into the nanoporous film was followed by protein diffusion into the nanoporous structure. The introduction of the nanoporous structure into the multilayered film resulted in increased loading and release of protein drugs [49].

**Fig. 3:**
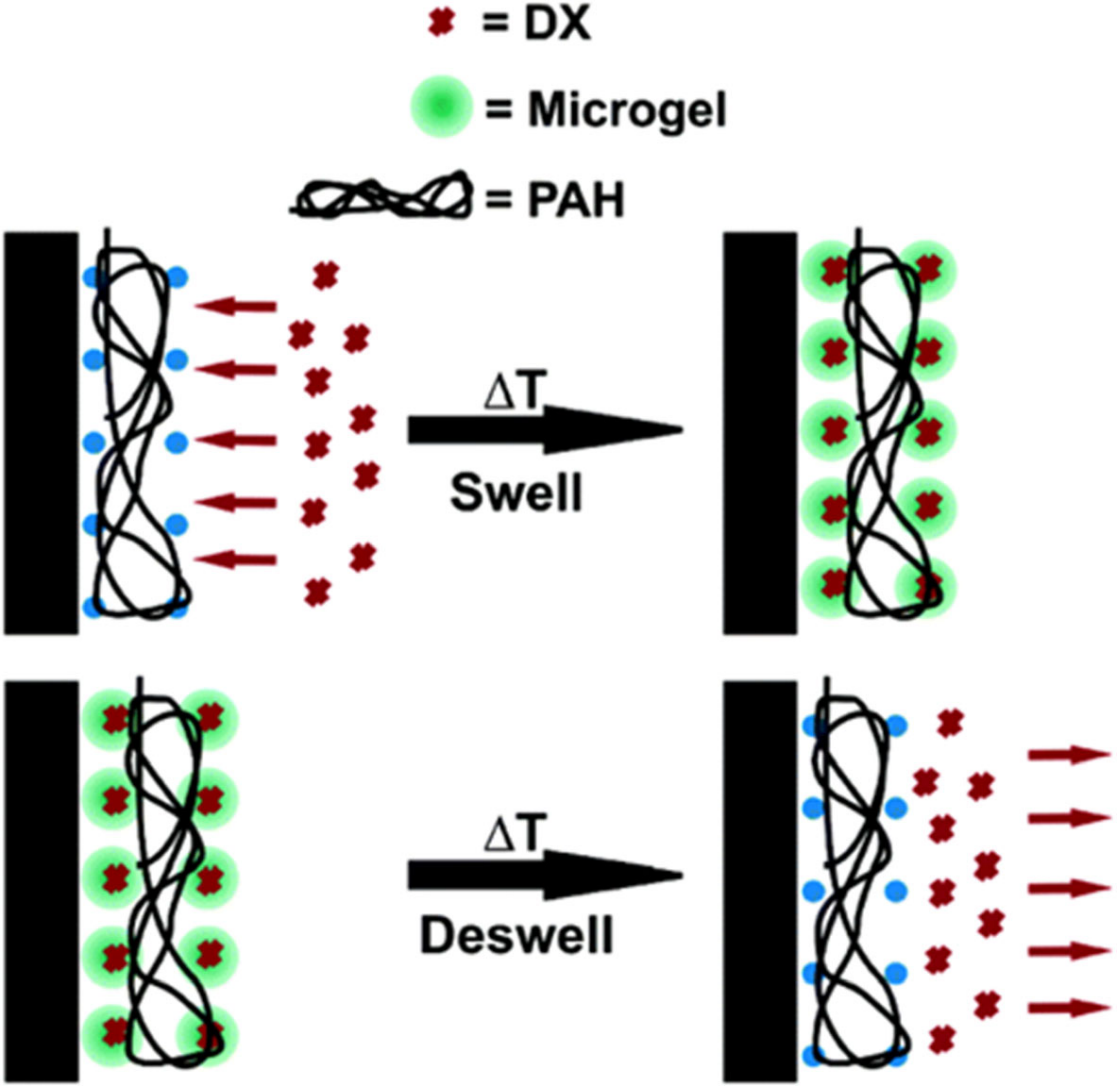
A schematic representation of the thin film configuration and the Dox loading/release mechanism. (Reprinted with permission from Ref. [50]. Copyright 2005, American Chemical Society)

The spin-assisted LbL assembly approach is also one of particular polyelectrolyte system. This technique was used to prepare thin films comprising thermo-responsive poly(N-isopropylacrylamide-co-acrylic acid) (pNIPAm-AAc) microgels by alternatively exposing a 3-aminopropyltrimethoxysilane (APTMS)-functionalized glass substrate to polyanionic pNIPAm-AAc microgels and polycationic poly(allylamine hydrochloride) (PAH). Doxorubicin (Dox) loading onto the microgel thin films was accomplished by changing the temperature of the film in a cyclic fashion between 25 and 50 °C, in an aqueous solution of doxorubicin, as shown in Fig. 3. The cyclic changes in temperature above the intrinsic LCST of the microgels in the films, allow modulation of both loading and release, where temperature-cycled films loaded Dox more efficiently than films that were not temperature-cycled [50].

### Preparing thin films using drugs as building blocks

Higher doses of drugs result in toxicity, making the LbL assembly method highly desirable for controlling drug release while simultaneously maintaining a drug concentration above the minimum inhibitory concentration (MIC). Shukla et al. [51]., used the LbL assembly method to fabricate vancomycin delivery coating. They used poly (β-amino ester) and vancomycin as a positively charged multilayer, and dextran sulfate, chondroitin sulfate (CS), and alginate as a negatively charged multilayer. In the study, they incorporated vancomycin to achieve a wide range of drug loading and controlled-release profiles, while maintaining the drug concentration above the MIC while avoiding toxicity at high doses [51, 52]. Min et al. [53]., fabricated LbL film coatings onto medical implants for osteomyelitis. Both gentamycin and BMP-2 are weakly positively charged and were used as polycations for preparing multilayered films, along with the negatively charged polyacrylic acid (PAA), via electrostatic interactions. The top layer consisted of [Poly1/PAA/GS(gentamycin sulfate)/PAA] tetralayers, which would inhibit bacterial biofilms, and the inner layer consisted of [Poly2/PAA/BMP-2/PAA] tetralayers, which would enhance bone formation [53].

**Fig. 4:**
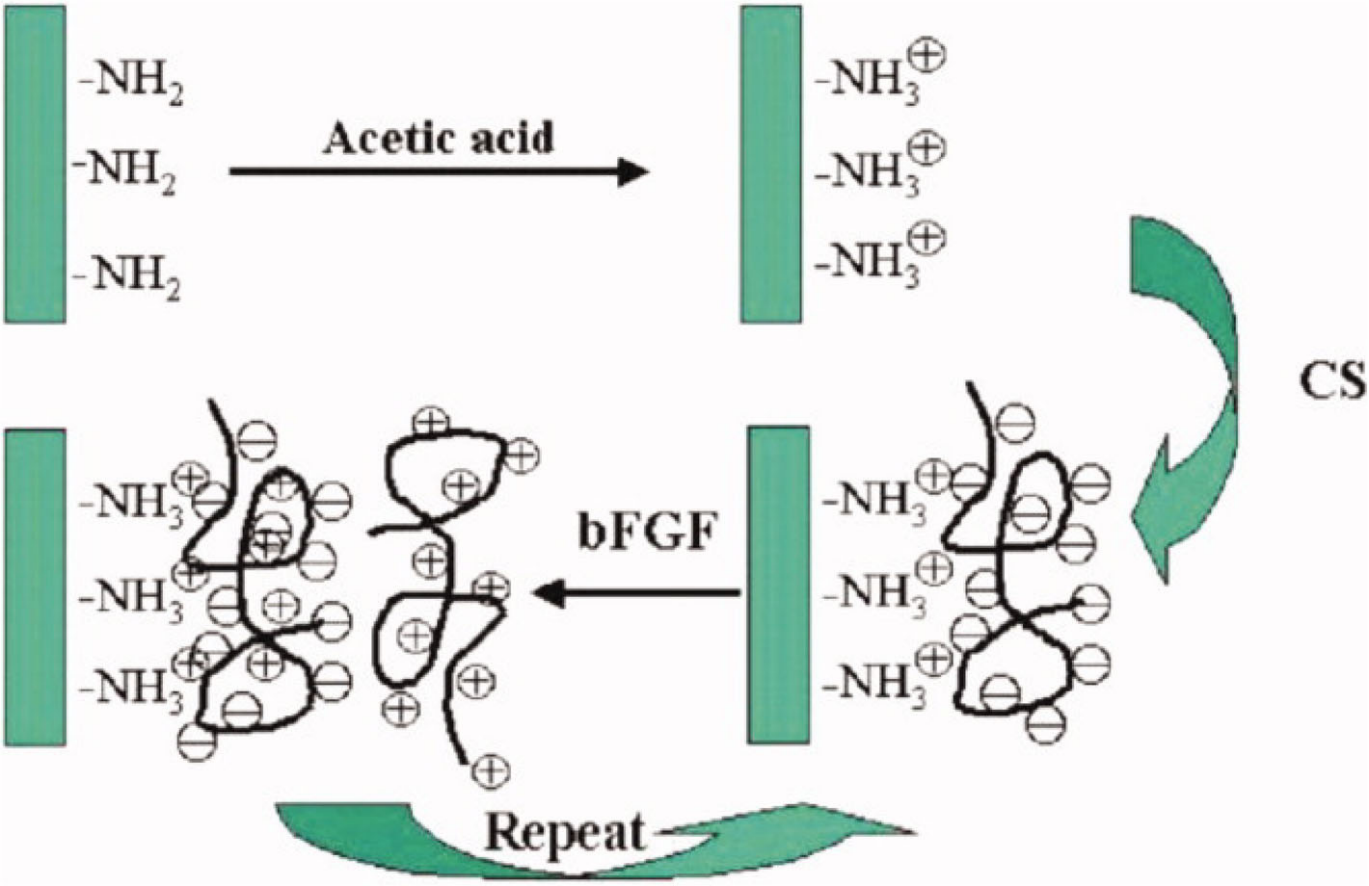
Schematic illustration of LbL assembly using chondroitin sulfate and bFGF. (Reprinted with permission from Ref. [54]. Copyright 2007, Wiley)

**Fig. 5:**
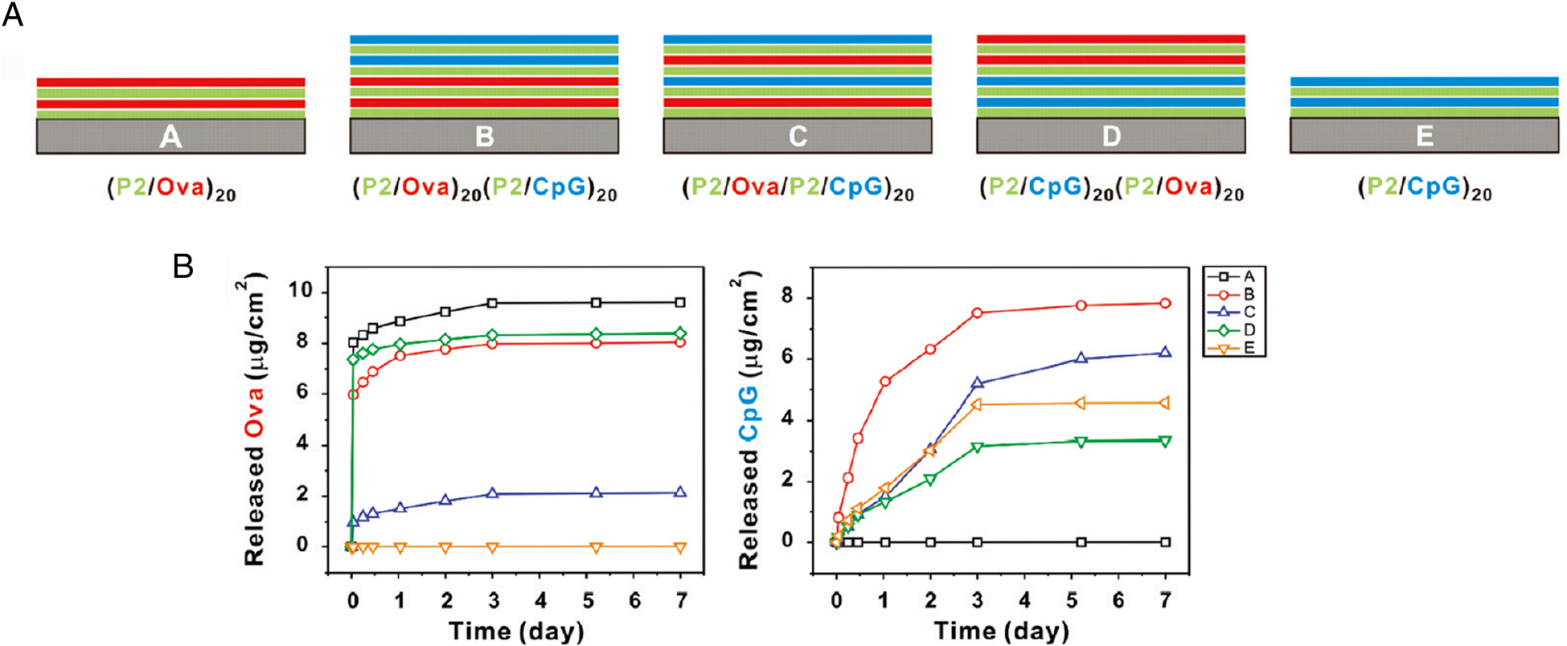
**a** Schematic architectures of antigen (ova) and adjuvant (CpG DNA) co-delivery films tested. **b** Loading and release amounts of ova and CpG from 5 different kinds of LbL films. (Reprinted with permission from Ref. [55]. Copyright 2009, American Chemical Society)

The incorporation of the basic fibroblast growth factor (bFGF) by LbL assembly is an example from the early developmental phases of LbL assembly, where the LbL method was used to enhance the stability and control the release of the growth factor. Ma et al. [54]., fabricated multilayered films of CS and bFGF. They prepared the multilayer film using the LbL assembly method, based on electrostatic interactions between the positively and negatively polyelectrolytes as show in Fig. 4. The CS/bFGF multilayer film exhibited an efficient release of bFGF, at a proper rate [54]. Su et al. [55]., studied the incorporation of hydrolytically degradable polymers and proteins by using the LbL assembly method. In their publication, ovalbumin and the oligonucleotide adjuvant molecule, CpG, were introduced into films using poly (β-amino ester) as shown in Fig. 5a. They also demonstrated that the loading amount of the drug depends on the film deposition order. In case of film B, the loading amount of CpG increased due to the under layers (P2/Ova film) (Fig. 5a and b). The underlying film led to the inter-diffusion of upper layer deposition, resulting in a higher loading efficiency [55]. Hong et al. [56]., constructed degradable multilayer films using 2 types of positively charged components, the poly(β-amino ester) and poly(L-lysine)(PLL). Ovalbumin was used as the model negatively charged antigenic protein. The study revealed that they could control protein releases with different mechanisms using a different polyions [56].

**Fig. 6:**
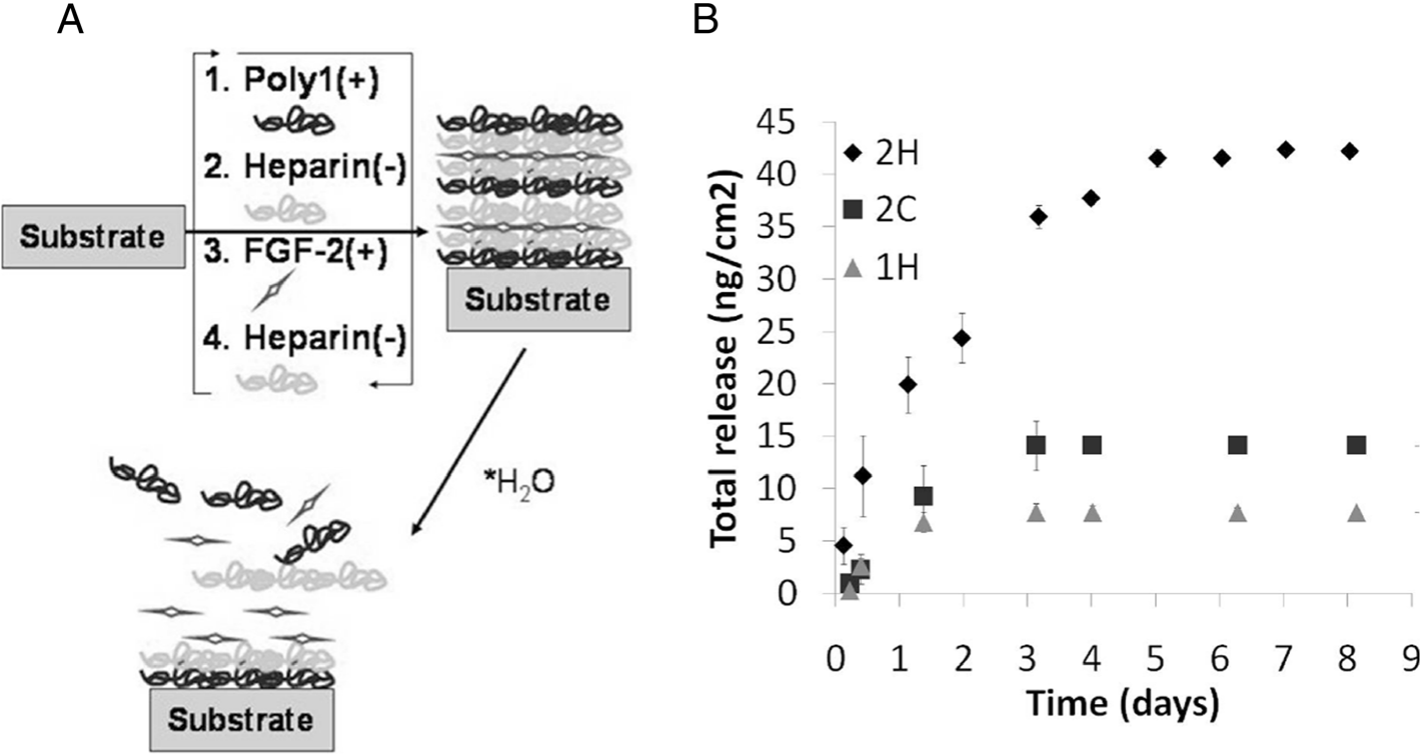
**a** Schematic illustration of the preparation of FGF-2 film using poly beta amino ester and heparin. **b** Total release and amount of incorporated FGF-2 from the 3 different films where 2H, 2C and 1H represent [Poly2/heparin/FGF2/heparin], [Poly2/chondroitin /FGF2/heparin] and [Poly1/heparin/FGF2/heparin]. (Reprinted with permission from Ref. [58]. Copyright 2010, American Chemical Society)

During LbL assembly, the amounts of drugs and their effectiveness can be controlled by manipulating various factors. The force of the interaction between drugs and building blocks affect the effectivity of drug incorporation. Helen F. Chuang et al. [57]., prepared antibiotic-incorporated films, using gentamicin as a building block. They chose hyaluronic acid as a polyanion and poly (β-amino ester) (Poly X) as a polycation. Specially, they used several different types of poly X, where X is the number of polymer repeat units. When large numbers of poly X were used, the gentamicin LbL film could grow rapidly due to increased hydrophobicity. However, the ratio of the amount of drug incorporated to the thickness (density of drug incorporation) was shown to decrease when large numbers of poly X were used, suggesting that a stronger electrostatic force within the film leads to higher loading amount of gentamicin [57]. Another researcher reported the preparation of fibroblast growth factor-2 (FGF-2)-loaded LbL films, using two kinds of poly (β-amino ester) and CS or heparin, as shown in Fig. 6a. To investigate the amount of incorporated FGF-2, they prepared 3 different kinds of LbL films; [Poly1/heparin/FGF2/heparin] (1H), [Poly2/heparin/FGF2/heparin] (2H) and [Poly2/chondroitin/FGF2/heparin] (2C). The study showed that the higher hydrophobicity of poly (β-amino ester), could allow interactions with FGF-2 via secondary interactions such as hydrophobic interactions, resulting in a higher loading amount of FGF-2. The biological interactions between heparin and FGF-2 also influence the loading efficiency (Fig. 6b) [58].

**Fig. 7:**
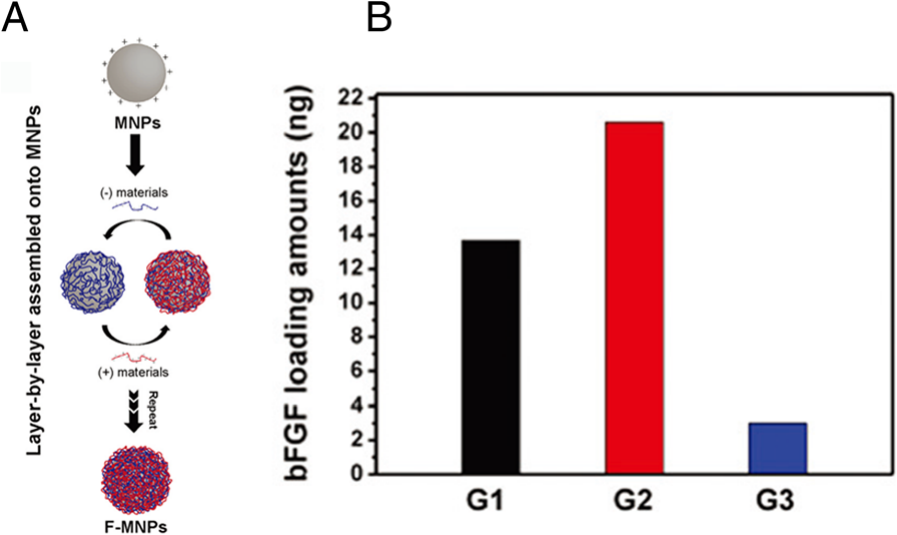
**a** Schematic illustration of the preparation procedure of bFGF-incorporated LbL films on magnetic nanoparticles. **b** Loading amount of bFGF onto three kinds of films represented as G1, G2 and G3 in the figure. The G films (growth factor films) included only bFGF and heparin for G1; bFGF, PLL and heparin for G2 and GO; and bFGF and heparin for G3. (Reprinted with permission from Ref. [59]. Copyright 2015, The Royal Society of Chemistry)

Additionally, the amount of drug incorporated into the films could alter, depending on the properties of the building blocks. D. Choi et al. [59]., prepared bFGF-incorporated LbL films for amine-functionalized magnetic nanoparticles (Fig. 7). In this LbL system, they selected the high molecular poly L-lysine (PLL), GO, and heparin as building materials. The loading amounts of bFGF in the three kinds of films were conducted due to the different properties of materials they used (Fig. 7). In case of PLL, the loading amount of bFGF was higher than that of the GO film, which is attributed to the higher molecular of PLL, which could load more bFGF within the film layer via inter-diffusion. However, GO being non-permeable, could not allow inter-diffusion into the film layer and blocked the outer release of bFGF, resulting in a low incorporation efficiency. The incorporation kinetics of drugs could be changed easily by manipulating the film assembly conditions [59].

### Using cargoes incorporated with drugs to prepare a film

Efficient drug delivery is designed keeping several factors in mind, including the cooperation of protecting drug, drug loading, reaching the target and drug release, which are all directly influenced by a drug’s molecular structure. An example includes some aldehyde-based drugs, which breakdown when exposed to the gastric juice in humans. This necessitates the protection of these drugs by a shell of a stable chemical structure, which should also be able to release the drug to the target, unlike the neutral camptothecin that cannot easily transfer drugs to their targets within the human body owing to its hydrophobic nature [60–62]. The driving force is very important to drug delivery systems, and some pharmaceutical drugs are difficult to assemble by the usual methods because there are no driving forces between the molecules or between molecules and loader [63, 64]. To solve these problems effectively, researchers have studied drug delivery using cargoes for modifying the fundamental properties, since encapsulation of the molecular drug cargo can protect the drug, preventing undesirable drug decomposition, and control the driving force. The therapeutic cargo has exhibited therapeutic efficacy in drug delivery systems [65].

#### The amphiphilic, block copolymer micelles cargo

**Fig. 8:**
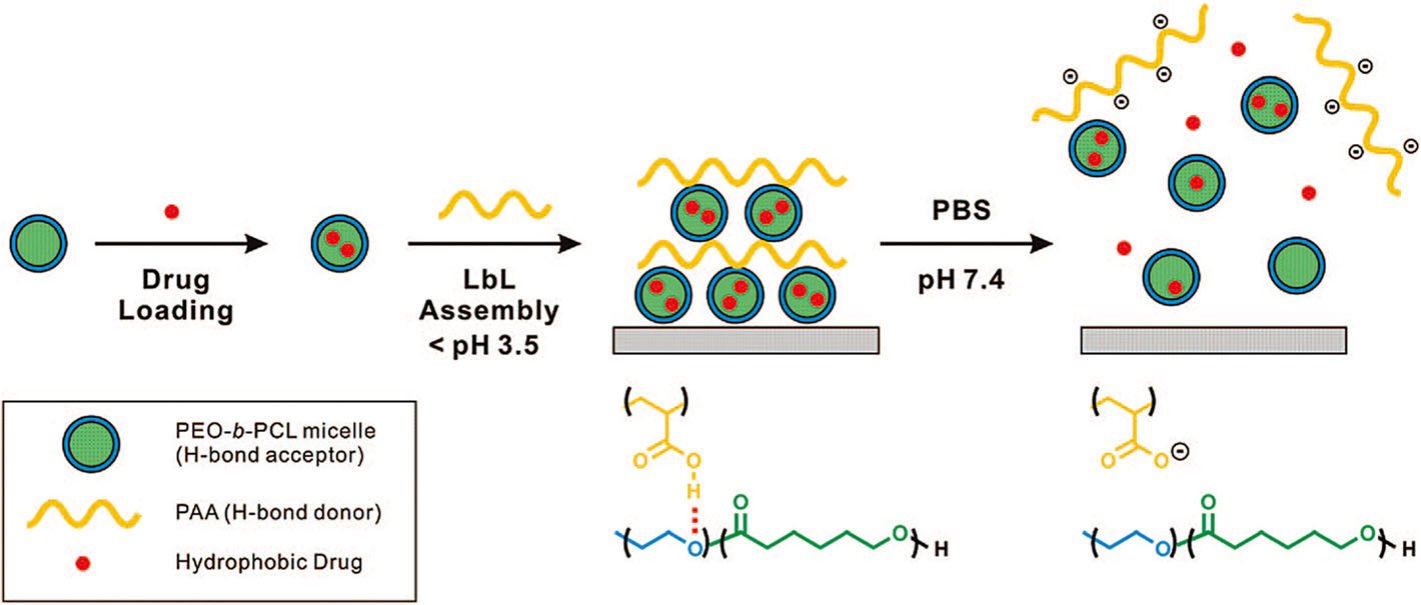
Schematic representation of hydrogen-bonds in the layer-by-layer assembly method used to synthesize BCM, which act as vehicles for hydrophobic drug delivery from surfaces. (Reprinted with permission from Ref. [33]. Copyright 2008, American Chemical Society)

**Fig. 9:**
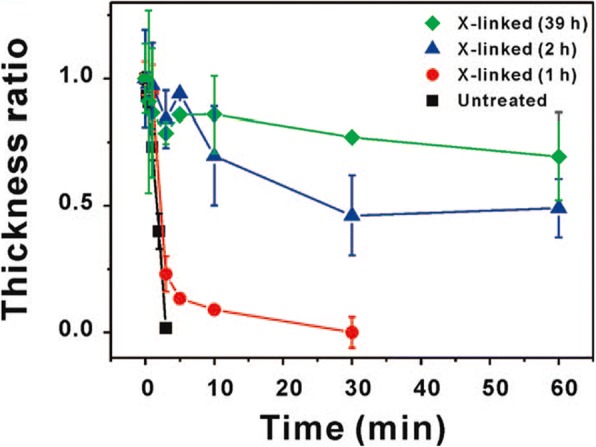
Change in film thickness as measured by profilometry. (Reprinted with permission from Ref. [33]. Copyright 2008, American Chemical Society)

Micelle formation can be controlled by the conditions of the solution such as pH, temperature, and ionic strength, which is suitable for drug delivery system. Micelles are sometimes used for the fabrication of multilayer thin films using LbL assembly [34]. The amphiphilic block copolymer micelles (BCM) can control assembly, and release materials for water-insoluble molecules because it allows researchers to control the degree of ionization by modifying the pH. B. Kim et al. [33]., reported the use of BCM for loading hydrophobic drugs within LbL films because it can perform drug loading and pH-assisted release via hydrogen bond interactions. As shown in Fig. 8, the author tried to fabricate the film using pH-sensitive hydrogen bonds between poly(acrylic acid) (PAA) and biodegradable poly(ethylene oxide)-block-poly(ε-caprolactone) (PEO-b-PCL) micelles. The pH-sensitive hydrogen bonds between PAA and PEO-b-PCL have an advantage in the LbL film, allowing the release of the hydrophobic drug from the micelle by simply modulating the pH. The authors attempted to control the rate of film deconstruction by introducing cross-links between the carboxylic acid groups in PAA to form anhydride linkages by applying heat, as shown in Fig. 9. The results indicated that the cross-links retard drug release into the surrounding medium to ensure a sustained drug release for several days [33].

**Fig. 10:**
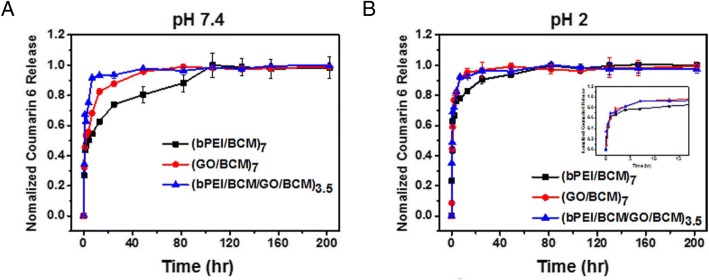
Coumarin-6 release profile from (bPEI/BCM), (GO/BCM), and (bPEI/BCM/GO/BCM) films in PBS buffer containing ethanol (2:1 PBS/EtOH) at **a** pH 7.4 and **b** pH 2. (Reprinted with permission from Ref. [32]. Copyright 2016, Nature)

Y. Han et al. [32]., reported the preparation of functional LbL films fabricated from PS-b-PAA BCM, encapsulating coumarin-6. Coumarin-6 was encapsulated in the micelle via hydrophobic interactions with PS. They focused on the mechanical properties of the LbL films, including thickness, roughness, and morphology, which can contribute towards disproportioned drug delivery. The authors introduced GO that has high mechanical property and is an attractive candidate for use with BCM. The film was fabricated using GO and branched polyethyleneimine (bPEI/BCM) as a buffer layer. The resulting GO/BCM film showed that film thickness can be effectively controlled by varying the pH of solution used in the fabrication process. Using these technologies, they optimized the release rate of coumarin-6 by controlling the pH of the solution, as depicted in Fig. 10. The development of the BCM-based drug delivery system represents a new approach for fabrication of therapeutic films with controlled drug release properties [29].

#### Protein and peptide cargo for encapsulation

**Fig. 11:**
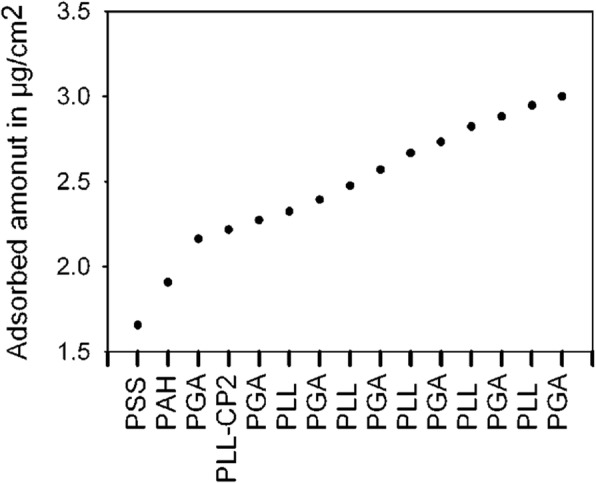
Graph showing that the deposited amount increases during PLL/PGA film preparation by addition of the PLL-CP2 layer. (Reprinted with permission from Ref. [66]. Copyright 2001, American Chemical Society)

Protein and peptide cargoes have been used in drug delivery systems due to their high order structures, including 2D nanonetworks, crystal meso phases and diverse functional groups. Above all, protein cargoes can store more biotins without losing bioactivities, than any other method. The protein cargo can selectively store the drug molecules through interactions with the protein amino acids. Chluba et al. [66]., reported the R-melanocortin derivative, where the protein cargo was covalently bonded with polylysines (PLL) that comprised the LbL thin film. The R-melanocortin derivative could, therefore, sustain the activity of the hormone for a long time in the multilayer thin film. As shown in Fig. 11, the synthesized PLL/R-melanocortin complex (PLL-CP2) was used in the LbL method, and this thin film could be better for storing free hormones [66].

Nadia et al. (2003)., suggested that protein cargoes could serve as building block components of multilayer films without the need for covalent bonding. Based on whether the protein has a negative or positive charge in aqueous solution, they used the Protein A (PA) which could bind with fragment c of immunoglobulin, and thus rendered bioactivities such as antitumor and anti-toxic properties to the protein cargo [67].

#### Mesoporous nano particles as a cargo in the LbL process

**Fig. 12:**
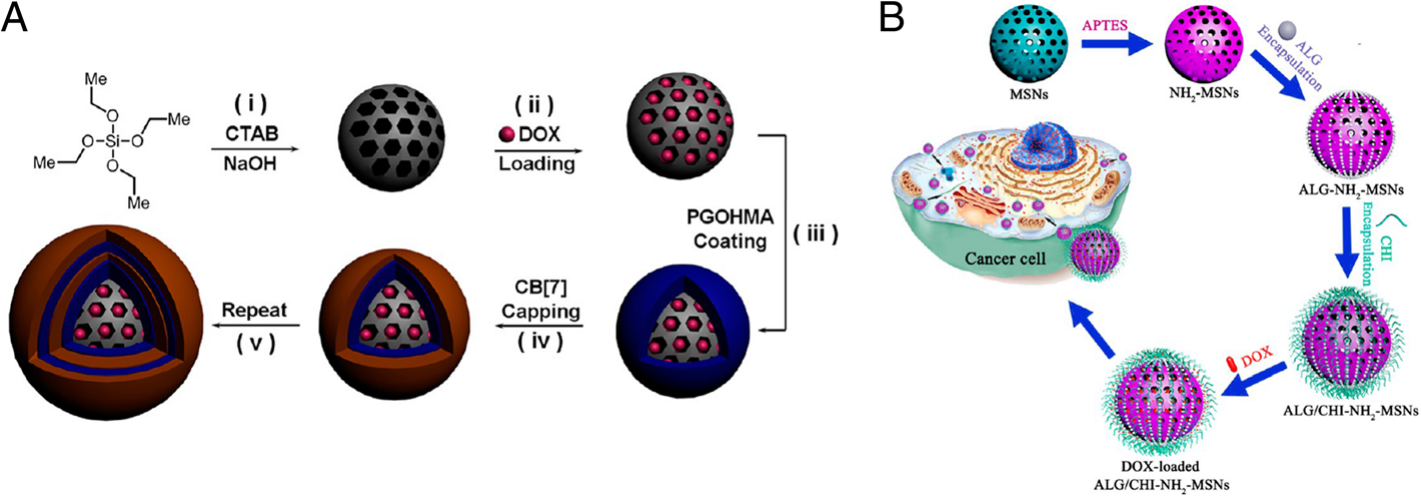
The scheme for preparing LBL-MSP with DOX **a** The method used by Qing-Lan Li et al. (Reprinted with permission from Ref. [68]. Copyright 2014, American Chemical Society) and **b** The method used by Wei Feng et al. (Reprinted with permission from Ref. [69]. Copyright 2014, American Chemical Society)

**Fig. 13:**
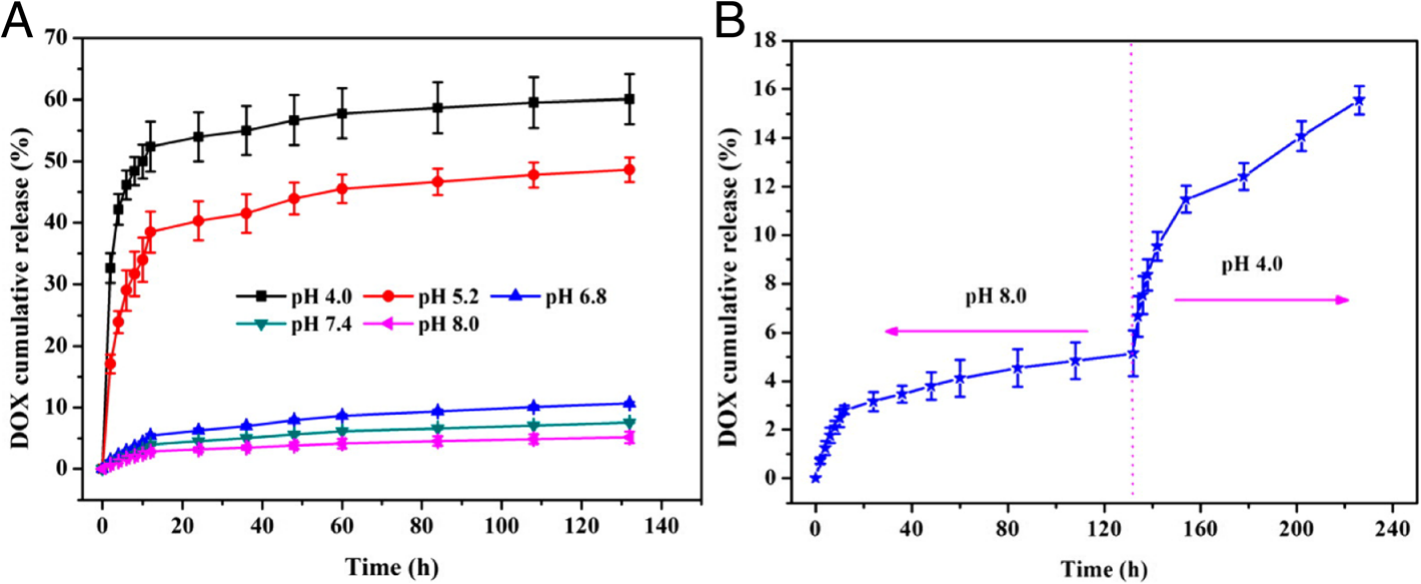
**a** The release profiles of DOX from DOX@PEM-MSNs at different pH values. **b** pH-controlled release of DOX from DOX @PEM-MSNs. (Reprinted with permission from Ref. [69]. Copyright 2014, American Chemical Society)

There are many approaches for better drug loading using inorganic mesoporous spheres having superior abilities to absorb both hydrophilic and hydrophobic molecules into their pores, and maintain high stability under the acidic conditions during the construction of hydrogen-bonded multilayers. Therefore, many researchers have been using the mesoporous spheres as LbL substrates, which can function as a drug cargo. Qi Li et al. [68]., reported that biocompatible LbL-coated silica macroparticles could release the encapsulated anticancer drug, doxorubicin hydrochloride (DOX), by pH stimuli or by competitive agents as shown in Fig. 12. Mesoporous particles can be used as drug cargoes and the multilayers on the particles can control drug release [68]. Wei Feng et al. [69]., also reported the use of mesoporous silica nanoparticles as an effective and biocompatible pH-responsive drug delivery system (Fig. 13). Comparing with the research reports of Qing-Lan Li et al., in this paper, after build the multilayer, DOX, drug was carried into the sphere.

## Application of thin film for targeted drug delivery

Among drug delivery systems, targeted drug delivery has been receiving considerable attention because of its therapeutic advantages of improving therapeutic action and decreasing side effects. Additionally, the LbL assembly method was used to build nano drug carriers, which have multiple functions, including enhanced drug stability, stimuli-responsive drug release, and dual drug release. These LbL-deposited multilayers are suitable for targeted drug delivery because drug release rate from multilayers can be regulated by manipulating the aforementioned factors. Especially, endogenous stimuli-responsive drug-releasing multilayers are responsive to factors such as pH [70], antigen [71], glucose [72], and lectin [73], and are of potential use in organ-targeted drug delivery. Moreover, LbL-deposited multilayers can include various materials such as antibodies, antigens, and antigen receptors, which offer ligand-directed targeting [74].

### Tumor targeting by LbL-assembled drug carriers

**Fig. 14:**
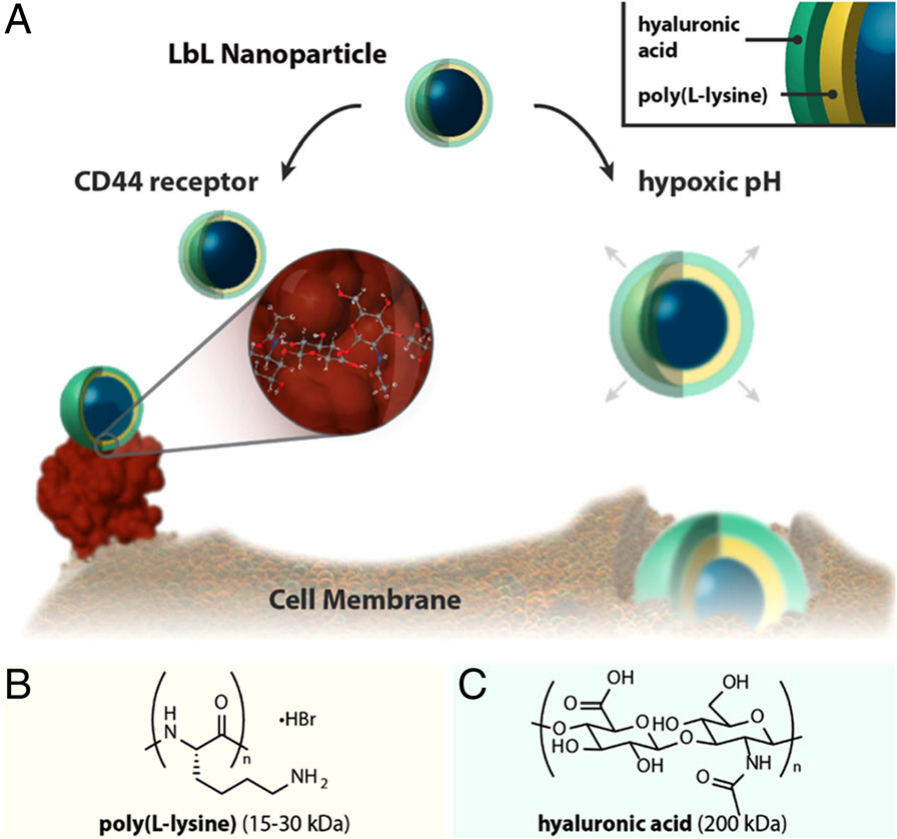
Hyaluronan Layer-by-Layer (LbL) nanoparticles actively target the hypoxic, low pH tumor by binding to the cancer stem cell receptor CD44. **a** Schematic illustrating bimodal tumor-targeted delivery. **b** Polycation and **c** Polyanion components of the LbL nanoparticle. CD44 protein structure in (**a**) is rendered from biological assembly 1 of PDB ID 1UUH. (Reprinted with permission from Ref. [75]. Copyright 2014, American Chemical Society)

Dreaden et al. [75]., described the tumor-targeting LbL nanoparticle, comprising poly(L-lysine) (PLL, 15–30 kDa) and hyaluronic acid (HA, 200 kDa) layers (Fig. 14) [75]. The hyaluronic acid selectively combines to overexpressed CD44 receptors on the surface of breast and ovarian tumor cells [76]. The LbL nanoparticle, which has HA on its outer shell, can therefore be used as a tumor-specific drug delivery carrier. Besides, the PLL/HA multilayer-coated nanoparticle swelled up and lost its negative surface charge in the pH of the hypoxic tumor tissue (pH ~ 6.0). These changes in the structure and properties of the LbL nanoparticle could increase nanoparticle uptake efficiency by tumor cells. The authors demonstrated that an enhanced hypoxic pH-mediated uptake of LbL nanoparticle in Hep G2 human hepatocyte cells. As a result, the cellular uptake of (PLL/HA)-coated nanoparticles (HA-NPs) increased 2.5-folds at a pH of 6, versus the physiological pH of 7.4. Additionally, CD44 targeting by the LbL nanoparticle was explained by measuring the MDA-MB-231 and -468 breast carcinoma cell migration, because the migration of cells decreased upon HA-NPs and CD44 receptor binding [77]. Consequently, the HA-NPs accumulated in the MDA-MB-231 breast carcinoma cells, 4.0 folds higher than the nanoparticles in the control study. Finally, they reported improved tumor targeting by HA-NPs in vivo using a mouse model.

**Fig. 15:**
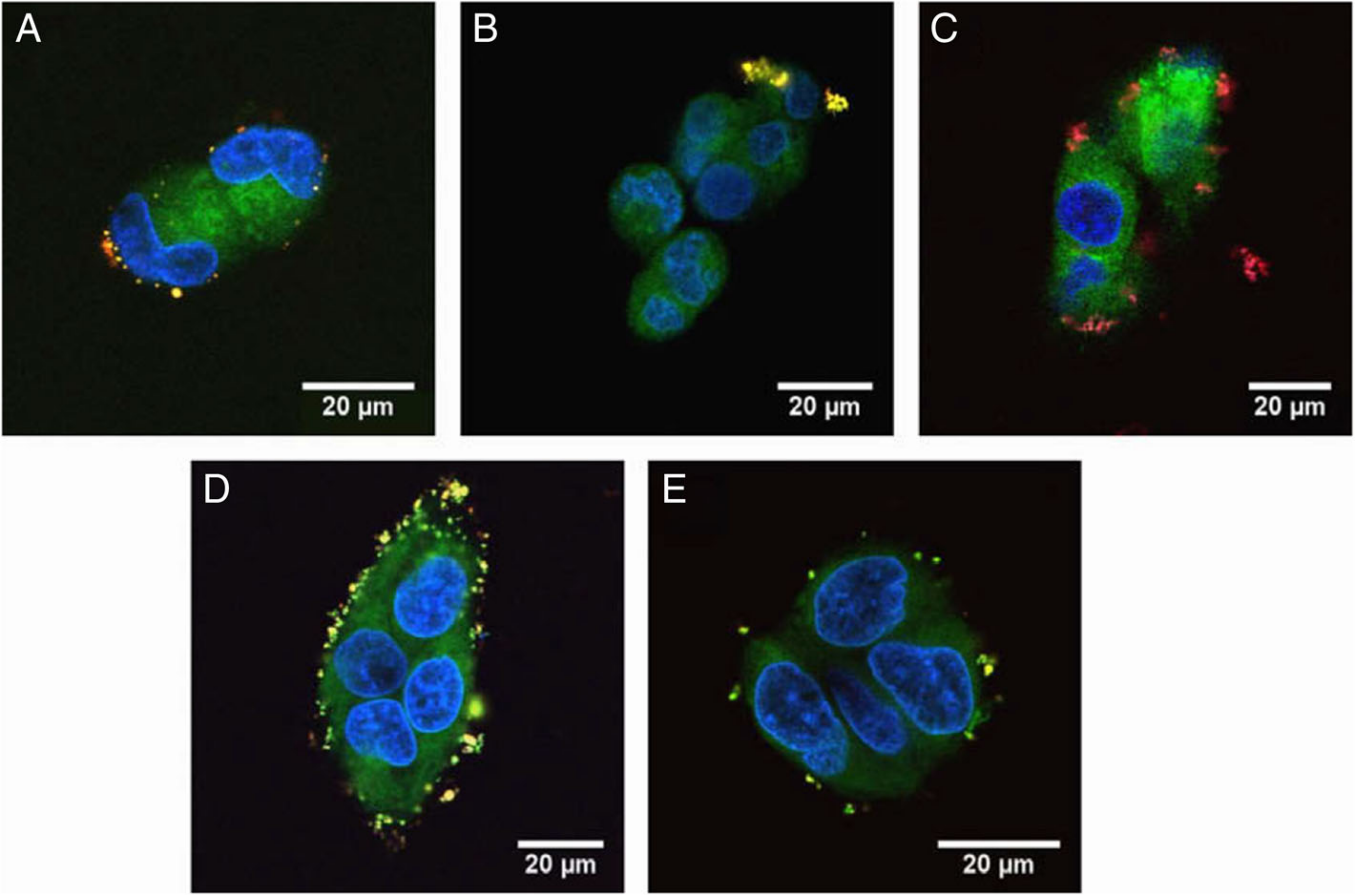
CLSM images of hepatocytes after co-culturing with: **a** bare NPs, **b** (Chi/Alg)2/Chi, **c** (Chi/Alg)2/Chi-FA, **d** (Chi/Alg)2/Chi-PEG–FA and **e** (Chi/Alg)3 covered NPs for 12 h. (Reprinted with permission from Ref. [78]. Copyright 2010, Elsevier)

The HepG2 cell-targeting poly (lactide-co-glycolide) (PLGA, 100 kDa) nanoparticle, coated with polyelectrolyte multilayer made up of chitosan (Chi, 100–300 kDa), alginate (Alg 25 kDa), and folic acid (FA)-conjugated poly (ethylene glycol) (FA-PEG) was designed by Zhou et al. [78]. The nanoparticle when coated with FA showed an increase in nanoparticle uptake by specific cancer cell lines, because the cancer cells overexpressed the folic acid receptor [79]. The Chi/Alg multilayer showed low interactions with albumin and low association with other cells and was used for antifouling coating. However, FA-PEG-bound (Chi/Alg) multilayer induced higher cell uptake and it can be used as a drug carrier for targeting tumors (Fig. 15).

### Targeting hepatocytes with LbL-assembled drug carriers

**Fig. 16:**
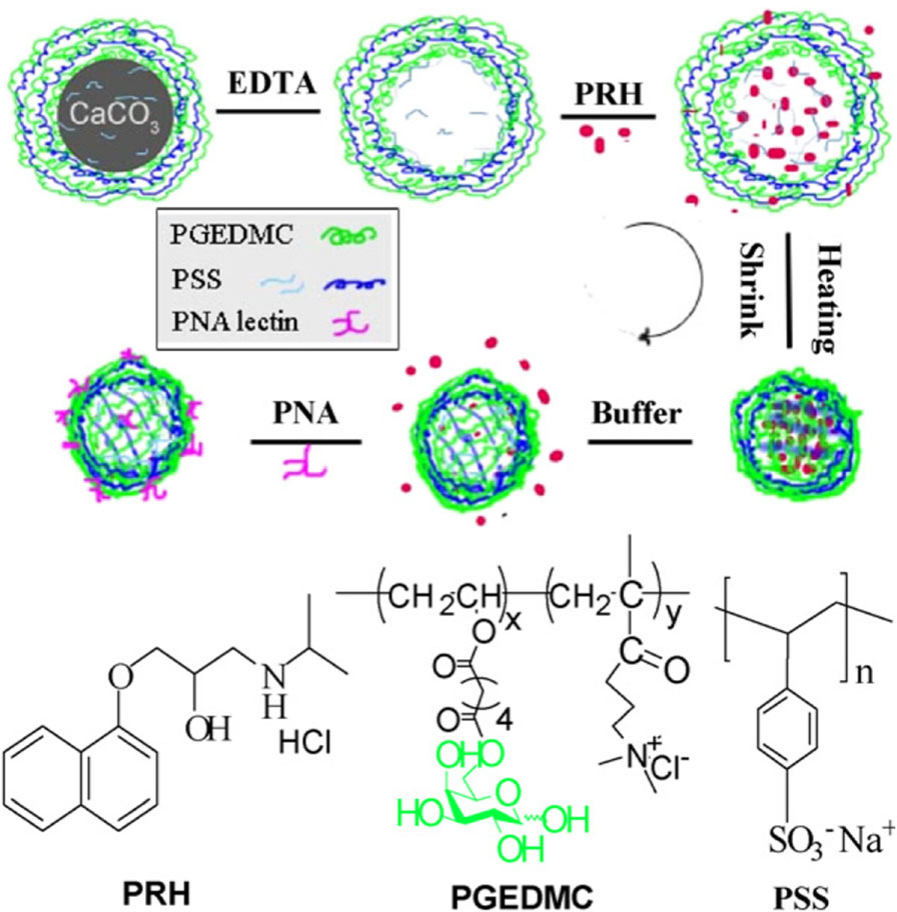
Schematic representation of enhanced drug delivery and reserved lectin-affinity by galactose-branched polyelectrolyte microcapsules after thermal treatment. (Reprinted with permission from Ref. [80]. Copyright 2008, Elsevier)

Zhang et al. [80]., introduced LbL-assembled multilayers as a drug delivery system for targeting hepatocytes, by using galactosylated polyelectrolyte (Fig. 16) [80]. D-galactose is well known for targeting hepatic cells because of its strong interaction with the asialoglycoprotein receptor expressed by the parenchymal cells of the liver [81]. The authors used the polycation poly(vinyl galactose ester-co-methacryloxyethyl trimethylammonium chloride) (PGEDMC, containing 19-mol% galactose residues), the polyanion poly(styrene sulfonate) (PSS), and the model drug propranolol hydrochloride (PRH). The size of the (PGEDMC/PSS)4.5 multilayered microcapsules decreased to 60% by thermal treatment at 70 °C. Thermal treatment also enhanced the loading, and ensured an effective controlled PRH release. PRH encapsulation capacity improved from 4.92 × 108 to 7.66 × 108 mol/capsule when the temperature was increased from 25 to 70 °C. Besides, the interactions between lectin and the galactose-containing microcapsules were maintained following heat treatment. The results showed that the heat-treated, (PGEDMC/PSS)4.5-multilayered microcapsule can be used as a drug carrier for targeting hepatocytes, due to its enhanced drug loading efficiency.

### Intestinal targeting by LbL-assembled drug carriers

**Fig. 17:**
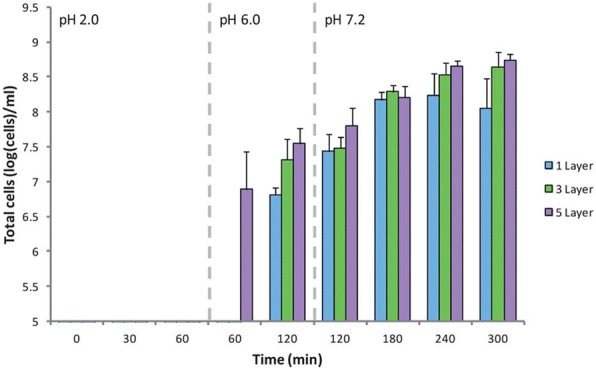
Release of *B. breve* from MCAMs under simulated gastrointestinal conditions. Limit of detection: 5 log(cells) per ml. Data given as the mean (*n* = 3) ± standard deviation. (Reprinted with permission from Ref. [82]. Copyright 2013, Royal Society of Chemistry)

Cook and co-workers produced alginate-chitosan multilayers, coated with alginate matrices for delivering probiotics to the intestines, by protecting them from the acidic pH of the stomach [82]. Commonly, probiotic bacteria lose their enzymatic activities in an acidic environment. Probiotic bacteria were encapsulated into the calcium cross-linked alginate matrix, to protect from denaturing [83]. The LbL-assembled multilayer matrix enhanced the viability of the model probiotic bacteria, Bifidobacterium breve (*B. breve*), and inhibited the release of *B. breve* into the gastrointestinal environment. The viability of free cell was < 3 log(CFU)/mL when exposed to the acidic environment of the stomach (pH 2.0), whereas that of the cells encapsulated in the 3-layer coated matrix was 8.84 ± 0.17 log(CFU)/ml under the same conditions. Since the alginate-chitosan multilayer remained stable at pH 2, and dissolved at the near-neutral pH region, the LbL-deposited alginate matrix could be used as a drug carrier for intestinal targeting (Fig. 17).

## Conclusions

In this research, the varied studies on Layer-by-layer (LbL) assembled multilayer thin films design for effective drug loading and targeting at desired sites have been reported. In LbL assembly technique, it is possible to fabricate excellent drug delivery carrier by selecting appropriate materials and driving forces because a wide variety of materials can be candidates for multilayer films in LbL assembly. There are three types of methods for preparing a drug-carrying multilayered film using LbL assembly. Methods included in the first type are direct loading of the drug into the pre-fabricated multilayer film. Second methods are preparing thin films using drugs as building blocks. In addition, the drugs are incorporated in the cargo so that the cargo itself can be used as the materials of the film. The appropriate designs of the drug-loaded film were produced in consideration of the release amounts and site of the desired drug. Furthermore, additional surface modification using the LbL technique enabled the preparation of effective drug delivery carriers with improved targeting effect. Therefore, the multilayer thin films fabricated by the LbL technique are a promising candidate for an ideal drug delivery system and the development possibilities of this technology are infinite.
